# Changes in Dynamics upon Oligomerization Regulate Substrate Binding and Allostery in Amino Acid Kinase Family Members

**DOI:** 10.1371/journal.pcbi.1002201

**Published:** 2011-09-29

**Authors:** Enrique Marcos, Ramon Crehuet, Ivet Bahar

**Affiliations:** 1 Department of Biological Chemistry and Molecular Modelling, IQAC-CSIC, Barcelona, Spain; 2 Department of Computational and Systems Biology, School of Medicine, University of Pittsburgh, Pittsburgh, Pennsylvania, United States of America; University of California San Diego, United States of America

## Abstract

Oligomerization is a functional requirement for many proteins. The interfacial interactions and the overall packing geometry of the individual monomers are viewed as important determinants of the thermodynamic stability and allosteric regulation of oligomers. The present study focuses on the role of the interfacial interactions and overall contact topology in the dynamic features acquired in the oligomeric state. To this aim, the collective dynamics of enzymes belonging to the amino acid kinase family both in dimeric and hexameric forms are examined by means of an elastic network model, and the softest collective motions (i.e., lowest frequency or global modes of motions) favored by the overall architecture are analyzed. Notably, the lowest-frequency modes accessible to the individual subunits in the absence of multimerization are conserved to a large extent in the oligomer, suggesting that the oligomer takes advantage of the intrinsic dynamics of the individual monomers. At the same time, oligomerization stiffens the interfacial regions of the monomers and confers new cooperative modes that exploit the rigid-body translational and rotational degrees of freedom of the intact monomers. The present study sheds light on the mechanism of cooperative inhibition of hexameric *N*-acetyl-L-glutamate kinase by arginine and on the allosteric regulation of UMP kinases. It also highlights the significance of the particular quaternary design in selectively determining the oligomer dynamics congruent with required ligand-binding and allosteric activities.

## Author Summary

Protein function requires a three-dimensional structure with specific dynamic features for catalytic and binding events, and, in many cases, the structure results from the assembly of more than one polypeptide chain (also called monomer or subunit) to form an *oligomer* or *multimer*. Proteins such as hemoglobin or chaperonin GroEL are oligomers formed by 2 and 14 subunits, respectively, whereas virus capsids are multimers composed of hundreds of monomers. In these cases, the architecture of the interface between the subunits and the overall assembly geometry are essential in determining the functional motions that these sophisticated structures are able to perform under physiological conditions. Here we present results from our computational study of the large-amplitude motions of dimeric and hexameric proteins that belong to the Amino Acid Kinase family. Our study reveals that the monomers in these oligomeric proteins are arranged in such a way that the oligomer inherits the intrinsic dynamic features of its components. The packing geometry additionally confers the ability to perform highly cooperative conformational changes that involve all monomers and enable the biological activity of the multimer. The study highlights the significance of the quaternary design in favoring the oligomer dynamics that enables ligand-binding and allosteric regulation functions.

## Introduction

The biological function of proteins is usually enabled by their dynamics under native state conditions, which, in turn, is encoded by their 3-dimensional (3D) structure. Unraveling this functional code has been the aim of many experimental and theoretical studies [1]–[9]. In particular the slow conformational dynamics of proteins in the micro-to-milliseconds time scale has been pointed out to be consistent with the changes in structure or domain/subunit movements observed between the substrate-bound and -unbound forms of enzymes [4]–[7],[10], and potentially limit the catalytic turnover rates of enzymes [11]–[14]. The quaternary structure of oligomeric proteins adds another layer of complexity to this code as the assembly of the subunits entails additional constraints while possibly inducing new types of collective motions. The structural hierarchy in oligomers indeed gives rise to a wide diversity of dynamical events [15]. For instance, in allosteric proteins, such as the paradigmatic hemoglobin [16], [17], the coupling between the internal dynamics of the subunits and the intrinsic ability of pairs of dimers to undergo concerted reorientations with respect to each other underlies the cooperative response to ligand binding [18]–[20]. Analysing the slow conformational dynamics thus emerges as a crucial step towards understanding the structure-function code in oligomeric proteins.

Two classical models have been broadly used in the literature to interpret the conformational changes observed upon ligand binding: the Koshland-Némethy-Filmer (KNF) model [21] where the ligand ‘induces’ a conformational change in the allosteric protein, in line with the classical induced fit model, and the Monod-Wyman-Changeux (MWC) model [22] where the ligand selects from amongst those pre-existing conformers accessible by the *intrinsic* dynamics of the 3D structure. The former is usually a stepwise process, while the latter is all-or-none. The experimentally observed structural changes appear to result from a combination of intrinsic and induced effects: the intrinsic dynamics of the protein prior to substrate binding is essential to enabling cooperative changes in structure, while induced motions, usually more localized, help optimize and stabilize the bound conformers [4], [23].

Protein-protein interfaces are usually characterized by their size, shape complementarity and hydrophobicity [24], [25]. The dynamics at the interfacial residues are usually given little attention, although the functional significance of the structural changes triggered by complex formation or oligomerization is widely recognized. The interface between subunits often plays a key role in mediating the activity of each monomeric subunit [25]. Protein-protein interactions provide, not only thermodynamic stability to the folded state of the subunit in the complex (or assembly), but also a new spectrum of collective motions. Furthermore, the oligomeric arrangement provides an efficient means of communication that may modulate allosteric regulation [19]. The present study focuses on the following questions: (1) Is the intrinsic dynamics of the component subunit modified by the oligomerization process, and if so, in which ways? (2) What is the role of interfacial interactions and overall contact topology in the functional dynamics of the oligomer and, in particular, in signal transduction or allosteric communication?

**Figure 1:**
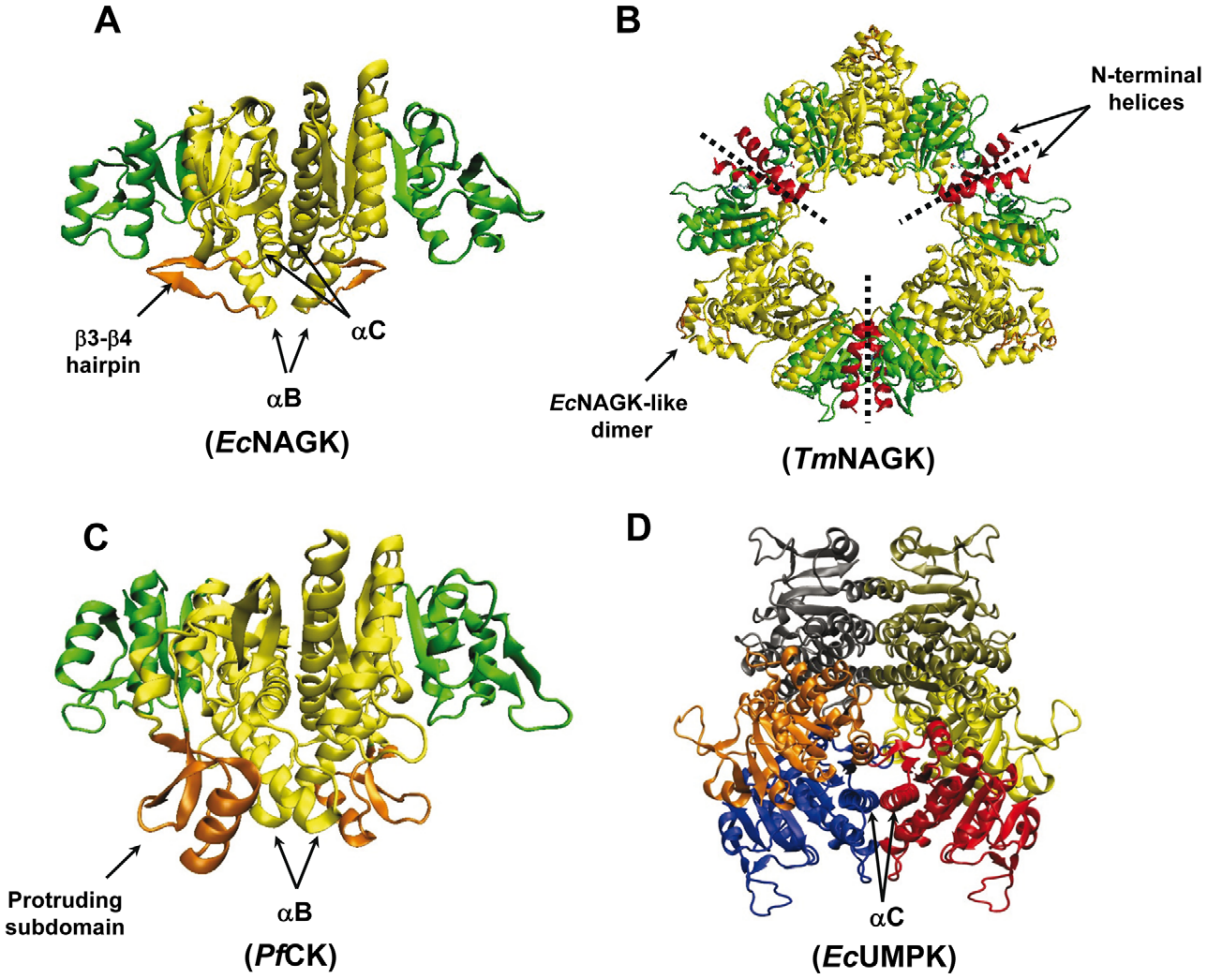
AAK family enzymes examined in the present study. (A) NAGK from *Escherichia coli* (*Ec*NAGK), (B) NAGK from *Thermotoga maritime* (*Tm*NAGK), (C) CK from *Pyrococcus furiosus* (*Pf*CK), (D) UMPK from *Escherichia coli* (*Ec*UMPK). Panels A, B and C show the ATP binding domains in green and N domains in yellow. The NAG-binding sites in *Ec*NAGK (β3–β4 hairpin) and the CK-binding site in *Pf*CK (protruding subdomain (PS) composed of the strand β5, helix D and hairpin β6-β7) are colored orange. The B helices of these two enzymes build part of the intersubunit surface and are very close to the N-domain binding sites. The N-terminal helices of *Tm*NAGK (red) interlink three *Ec*NAGK-like dimers (delimited by dotted lines). This hexameric enzyme is indeed regarded as a trimer of *Ec*NAGK-like dimers. The UMPK is colored by chains. αC helices indicated in panels A and D highlight the difference in the assembly of the monomeric subunits between the two structures.

The effect of multimerization on protein dynamics is investigated here in the context of the Amino Acid Kinase (AAK) family of enzymes. Members of this family have different degrees of oligomerization (Figure 1). Rubio and co-workers have significantly contributed to our current knowledge of this family of enzymes: they have resolved the X-ray structures of most family members [26]-[33] and suggested a shared mechanism of action on the basis of their sequence and folding similarities [28]. This mechanism was elucidated by our recent computational study of the softest modes of motion intrinsically accessible to different members of the AAK family of proteins [34].

The most exhaustively studied member of the AAK family is *N*-acetyl-L-glutamate kinase (NAGK) (Figure 1A). NAGK phosphorylates the amino acid *N*-acetyl-L-glutamate (NAG) in the bacterial route of arginine biosynthesis. In many organisms, NAG phosphorylation is the controlling step of the route, as NAGK is feedback inhibited by the end product arginine. Rubio and co-workers [30] characterized the structures of two hexameric NAGKs (from *Thermotoga maritima* (Figure 1B) and *Pseudomonas aeruginosa*) that are cooperatively inhibited by arginine [35]. In *Escherichia coli*, NAGK (*Ec*NAGK) is homodimeric and arginine-insensitive (Figure 1A). Indeed, several studies have proven that the hexameric arrangement is a requirement for the cooperative inhibition by arginine [30], [36]. The distinctive feature of this biosynthetic route in bacteria is that it produces *N*-acetylated intermediates, in contrast to mammals that yield non-acetylated intermediates. This turns NAGK into a potential target for antibacterial drugs by selective inhibition. Another member of the AAK family is carbamate kinase (CK; Figure 1C). CK catalyses the formation of ATP from ADP and carbamoyl phosphate (CP; a precursor of arginine and pyrimidine bases), and undergoes a substantial change in its structure upon substrate binding [37]. A third member is the hexameric UMP kinase (UMPK) (Figure 1D). UMPK catalyzes the reaction ATP + UMP 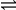 ADP + UDP to yield uridine diphosphate (UDP). It is involved in the multistep synthesis of UTP, being regulated by the allosteric activator GTP and inhibited by UTP itself. Its monomer fold is very similar to the rest of family members, but presents a strikingly different assembly of the subunits that has not been explained so far.

Notably, while the AAK family members do not exist in monomeric form, they share the same monomeric fold. This commonly shared monomeric fold is stabilized by oligomerization. The selection of a common monomeric fold in different oligomers suggests that that particular architecture possesses structure-encoded dynamic features that are exploited for enzymatic activity in oligomeric state. It is essential to analyze what the intrinsic dynamics of the monomeric units are, and to what extent, if any, they are maintained in the oligomeric state, or how they are coupled to, or complement, the dynamics of the biologically active (oligomeric) state. Calculations are thus performed for the monomeric fold alone as well as the monomer in the context of different oligomeric states, and the intact oligomers. As will be shown below, the oligomers do maintain some intrinsic dynamic features of the monomeric units, while the different assembly geometries of the monomers give rise to global motions uniquely defined for the particular oligomerization states. The method of analysis presented here is applicable to any protein that functions in different multimeric states. The effect of oligomerization on the dynamics of the component subunits can be experimentally examined provided that the protein exists in monomeric and different oligomeric states, which, in turn, may be controlled by environmental conditions [38] and few mutations at the protein surface [39]. However, such studies may be challenging in practice, and a computational examination emerges as an alternative promising tool.

The most collective movements of biomolecular systems, also called the *global* modes of motions, can be determined using Elastic Network Models (ENMs) in conjunction with Normal Mode Analysis (NMA) at very low computational cost. A wealth of studies have shown the robustness of the global modes predicted by the ENMs (e.g., by the anisotropic network model, ANM [40], [41]) and their close relevance to experimentally observed structural transitions related to ligand binding [4]-[6], [10], [18], [41]–[46], or to the essential modes extracted from converged molecular dynamics (MD) simulations [47]–[49]. The global modes are the low-frequency modes extracted from NMA, also referred to as *slow* modes. They correspond to large-amplitude motions taking place at long timescales (e.g. microseconds to milliseconds); and they are also called *soft* modes due to their lower energy cost associated with a given level of fluctuation away from the equilibrium state, compared to other modes. Given their robustness and efficiency, ENMs are uniquely suited for exploring the collective motions and allostery in oligomers. Previous such studies have highlighted the significance of multimeric arrangement in defining the collective dynamics [50]–[54].

The present study adds new evidences to the role played by multimerization in defining functional dynamics. First, we contrast the low-frequency modes favoured by the *Ec*NAGK and *Pf*CK monomers to those preferentially selected by the corresponding dimers. Secondly, the modes of the monomeric and dimeric components of hexameric *Tm*NAGK are compared to those collectively accessible in the hexameric form. Third, a detailed analysis of the softest modes accessible to the *Ec*UMPK dimeric form is presented to shed light onto the role played by different dimeric assemblies found in the AAK family in selecting the functional motions of the family members. Overall, the different designs of interfaces and assembly geometries observed among the members of the AAK family are shown to practically define the collective modes that are being exploited by the oligomers for achieving their particular activities, including substrate binding and allosteric regulation.

## Results/Discussion

### Soft modes intrinsically accessible to the monomer are selectively utilized or obstructed in compliance with the specific substrate-binding properties of the dimer: *Ec*NAGK vs *Pf*CK

**Figure 2:**
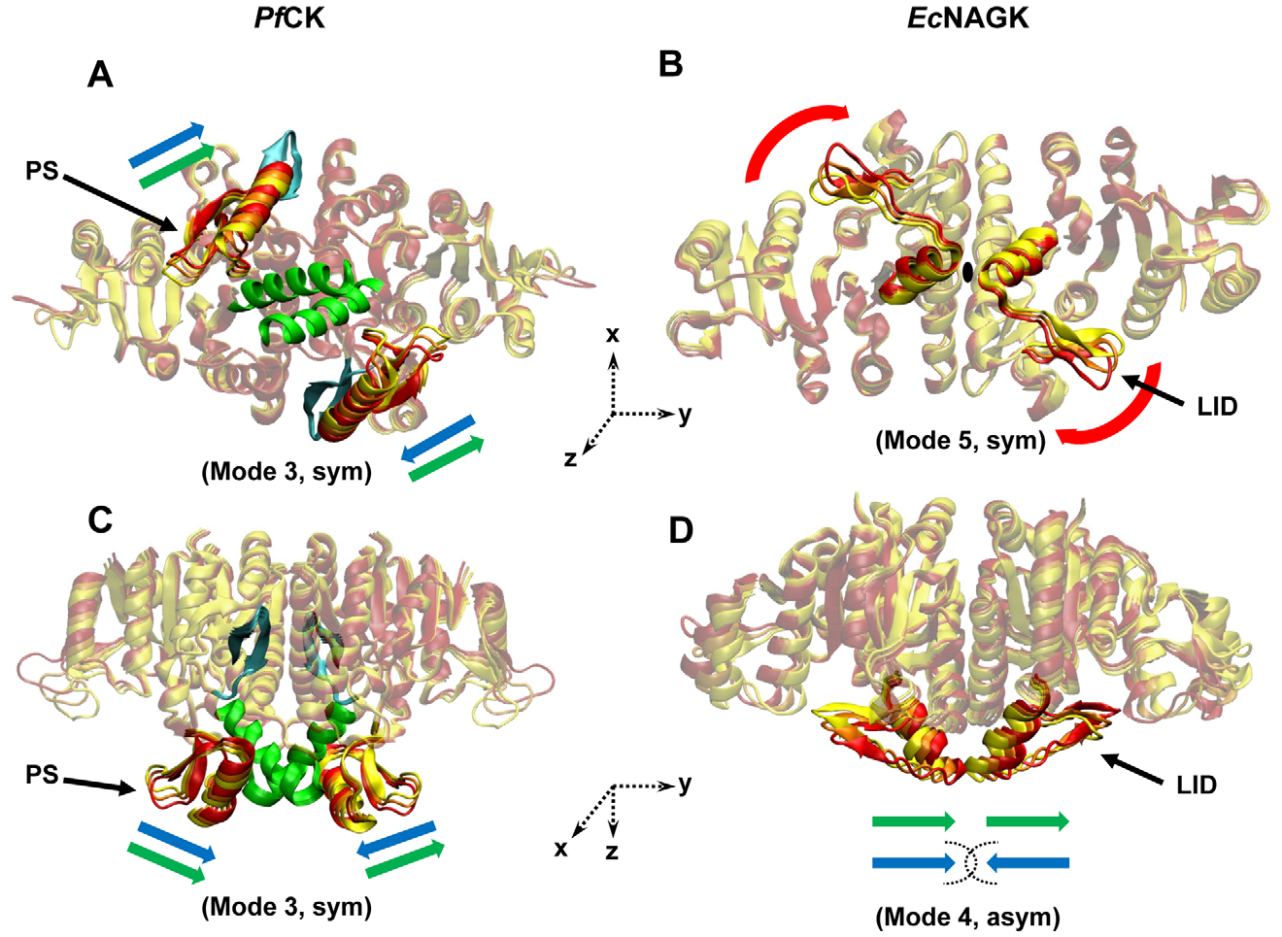
Dynamics of the substrate-binding sites on *Pf*CK and *Ec*NAGK. Panels A and C are bottom and lateral views of *PfCK*. Panels B and D are bottom and lateral views of *Ec*NAGK. Each panel shows different conformations (in yellow, orange and red) along a given mode (the number and the symmetry are specified in parenthesis). The structural elements involved in substrate-binding are highlighted by the brighter colors: PS in *Pf*CK and the β3–β4 hairpin (lid) in *Ec*NAGK. In *Pf*CK, helix B (green) and β10–β11 hairpin (cyan) are key structural elements at the dimer interface. In *Ec*NAGK, helix B, which is connected to the β3–β4 hairpin, is also highlighted. Green and blue arrows indicate the mechanisms of the modes that induce asymmetric and symmetric opening/closure at the substrate-binding site, respectively. In panels A and C, the green arrows show that the corresponding asymmetric movements of PSs are also allowed (4^th^ mode). In panel B, the black ellipse displayed at the interface shows the axis of rotation (z-axis), normal to the plane of the figure. The blue arrows in panel D show that the opposite movement of β3-β4 hairpins is not allowed due to the steric clashes. For better visualization of these modes see Videos S1, S2, S3 and S4.

How does the intrinsic dynamics of the monomeric subunits affect the oligomerization process or *vice versa*? To what extent the intrinsic dynamics of the monomers prevail in the oligomers? Or to what extent they are perturbed by oligomerization? To analyse these issues, we have first compared the low-frequency ANM modes of the dimeric *Pf*CK and *Ec*NAGK with those of their respective monomers. The two enzymes exhibit close structural similarities (Figure 2). Their sequence identity is 24%, and their ATP-binding site and catalytic sites exhibit similar structural features. In fact, our previous comparative analysis of their collective dynamics showed that the slowest three ANM modes, which essentially modulate the opening/closure of the ATP-binding site, are commonly shared between these two enzymes; and they yield an overlap of 0.75 with the experimentally observed reconfiguration from open to closed state of NAGK [34].

The main structural difference between *Pf*CK and *Ec*NAGK, on the other hand, resides in their amino acid substrate binding site, and here we focus on the softest modes that control those sites. In *Ec*NAGK, the β3–β4 hairpin serves as the lid of the NAG binding site and interlinks helices B and C, which are key components of the interface (Figure 1A); in *Pf*CK (Figure 1C), a subdomain protruding away from the interface serves as the lid of the CP binding site. This subdomain (PS) is formed by the strand β5, helix αD and hairpin β6–β7. Both lids exhibit significant conformational changes closely linked to substrate binding, as shown by the crystallographic studies performed by Rubio and co-workers [27], [32]. Among the ANM modes that affect the substrate-binding sites, those simultaneously leading to closure/opening of the substrate-binding site in both subunits will be called symmetrical modes, and others, asymmetrical (Figure 2).

### Description of the modes

In *Ec*NAGK, the symmetrical opening/closure of the substrate-binding sites is enabled by the 5^th^ mode (red arrows in Figures 2B and 2D; see Video S1), whereas the corresponding asymmetrical motion takes place in the 4^th^ (green arrows) mode (Video S2). Note that our previous work [34] showed that ANM modes 1–3 were instrumental in accommodating the structural changes at the ATP-binding site, but had practically no effect on the NAG-binding site. This nicely illustrates how the enzyme takes advantage of different types of motions accessible to its native structure for achieving different types of functional motions. In mode 5, the two β3–β4 hairpins (Figure 1A), the lids of the NAG-binding sites, undergo an almost rigid-body rotation about the dyadic (z-) axis of the molecule while the ATP binding domains undergo smaller but coupled anticorrelated rotations. On the other hand, the asymmetrical motion (mode 4) induces a translation along the *y* axis in both lids, along with the C-terminal part of the two helices B which are connected to the lids. No symmetric opening/closing of the lids is observed about the *y*-axis because these movements would be prohibited by steric clashes between the two B-helices (blue arrows in Figure 2D). Rotational motions about the z-axis, on the other hand, are favored by the overall architecture of the dimeric enzyme. Indeed, tight interfacial interaction between the two B-helices is considered to be a key element for the stability of the dimer [28]. The interfacial region thus coincides with the central hinge site that mediates the opening/closing of the two monomers. This example emphasizes the effect of inter-subunit surface and topology on the character of the movements allowed/prohibited, or selected, in the oligomer.

As to *Pf*CK, the two substrate-binding subdomains are able to undergo both symmetric (1^st^ and 3^rd^ mode; see Video S3) and asymmetric (4^th^ mode; see Video S4) motions because these two subdomains protrude away from the interface and their rotational rigid-body motions are not constrained by potential clashes between the adjacent B-helices. Indeed, the motion is parallel, rather than normal, to the plane defined by the two B-helices, and the two B-helices remain tightly packed and almost immobile in these modes. Notably, the global fluctuations of two PSs on *Pf*CK dimer appear to modulate the access to the substrate-binding sites, suggesting a role in mediating substrate-binding.

### Comparison between the monomer and dimer dynamics

The selection of particular modes by *Ec*NAGK for achieving its specific functions (e.g., modes 1 and 3 enabling ATP-binding; and mode 5, substrate binding) [34] raises the following question: is the rotation of the hairpins an acquired mode of motion originating from the topology of the dimer interface and not accessible to the monomer? Or, is it an intrinsic dynamical ability of the monomer that is conserved and exploited in the dimer? To address this issue, we compared the modes obtained for the isolated monomer with those of the monomer in the dimer, using the subsystem/environment coupling method described in the Methods. The monomer is the *subsystem*, and the second monomer stands for the *environment* in this case. For the sake of clarity, herein the modes that include the coupling to the environment are indicated with a superscript, i.e., monomer^(dimer)^ refers to the behaviour of the monomer within the dimer.

**Figure 3:**
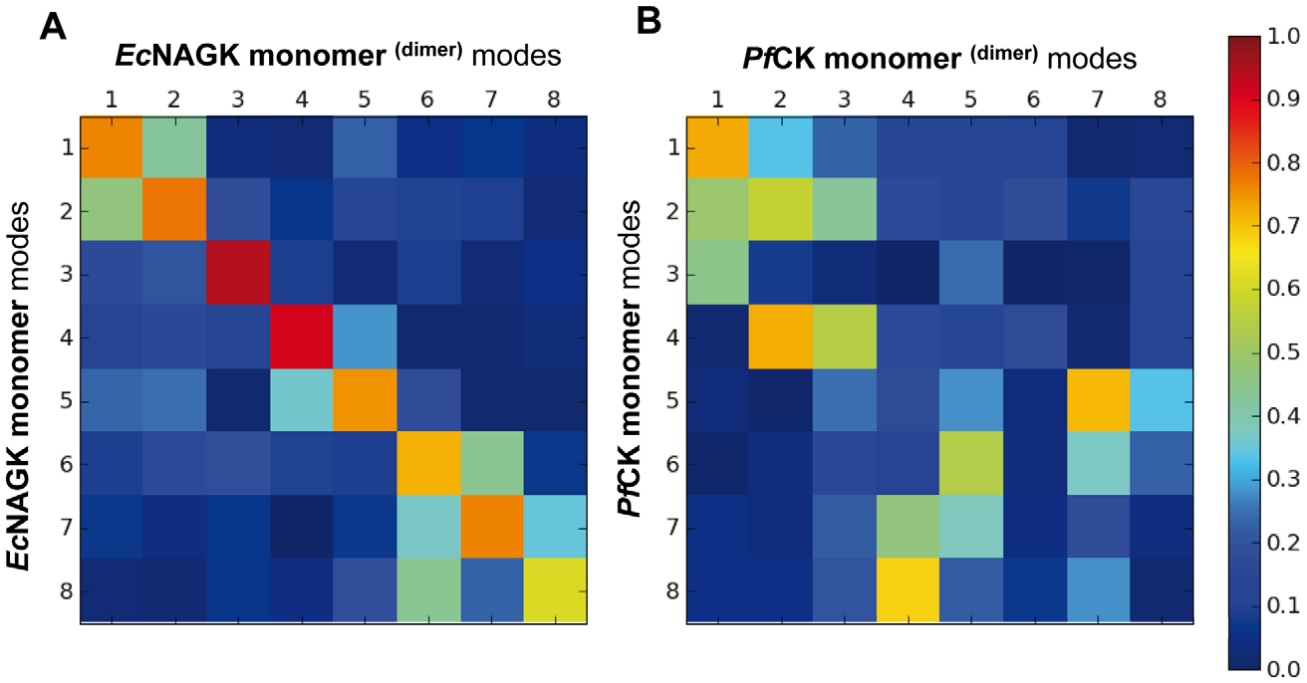
Comparison of the global dynamics of *Ec*NAGK and *Pf*CK monomers in the dimer with those of the isolated monomeric components. Overlaps between the eight slowest modes of the monomers and dimers of (A) *Ec*NAGK and (B) *Pf*CK are shown in the heat map. Dimerization has minimal effect on the intrinsic global dynamics of *Ec*NAGK, while that of *Pf*CK appears to be more strongly affected, presumably due to its larger intersubunit interface.

The results are presented in Figure 3 (and Supplementary Tables S1 and S2). Therein the overlaps between the eight lowest-frequency modes accessible to the monomer in the isolated state (y-axis) and within the dimer (x-axis) are displayed for *Ec*NAGK (panel A) and *Pf*CK (panel B), and Tables S1 and S2 lists the corresponding values. The orange-red entries along the diagonal in panel A demonstrate that the modes intrinsically accessible to the *Ec*NAGK are closely maintained in the dimeric enzyme. Notably, both the order of the modes (i.e., their relative frequency and size, as defined by the respective eigenvalues), and their shapes are closely conserved.

The picture is different in the case of the *Pf*CK dimer (panel B). While in *Ec*NAGK all of the top-ranking seven modes are maintained with an overlap of 0.70 or above, in *Pf*CK significantly fewer global modes favored by the isolated monomer are maintained, and with a weaker correlation and reordering of the modes. Thus, the *Pf*CK monomer dynamics is strongly affected by dimerization. Examination of the individual modes showed that the monomer modes that induce high fluctuations at particular secondary structural elements such as the helix B and the β10–β11 hairpin (shown in cyan in Figures 2A and C) are practically absent in the dimer. As shown in Figure 2 these are key elements at the intersubunit interface, and dimerization imposes high constraints quenching their motion. The intersubunit surface of *Pf*CK (2453 Å^2^) [27] is remarkably bigger than that of *Ec*NAGK (1279 Å^2^) [28]. This higher surface area, and ensuing closer association of the two monomers, may be partly responsible for the larger perturbation of the intrinsic dynamics of the monomer upon dimerization in *Pf*CK, compared to *Ec*NAGK.

Figure 2 and videos S3 and S4 in the Supporting Information demonstrate that the global motions preferentially undergone by the two PSs in the *Pf*CK dimer induce conformational changes near the substrate-binding site; and Figure 3 shows that the global dimer dynamics departs from that of the isolated monomers. So, dimerization promotes in this case collective motions that affect substrate recognition and/or binding. The PS has been proposed to have evolved, together with the intersubunit interface, to play a key role in the specificity of CK for its substrate carbamate, as opposed to more abundant analogues, i.e., acetate, bicarbonate or acetylphosphate [37]. This conjecture originally inferred from the examination of crystal structure alone is supported by our examination of *Pf*CK dynamics. ANM global modes clearly indicate the ability of the PS to undergo movements toward the substrate-binding site, and the enhanced mobility at this particular region may indeed underlie the adaptability of CK to bind its substrate.

### Conservation and creation of functional modes: the hexameric *Tm*NAGK

The next case we studied is the hexameric form of the NAGK enzyme from *Thermotoga maritima* (*Tm*NAGK). The higher degree of multimerization of *Tm*NAGK will permit us to contrast the dynamics of the whole enzyme with those of its dimeric and monomeric components.

**Figure 4:**
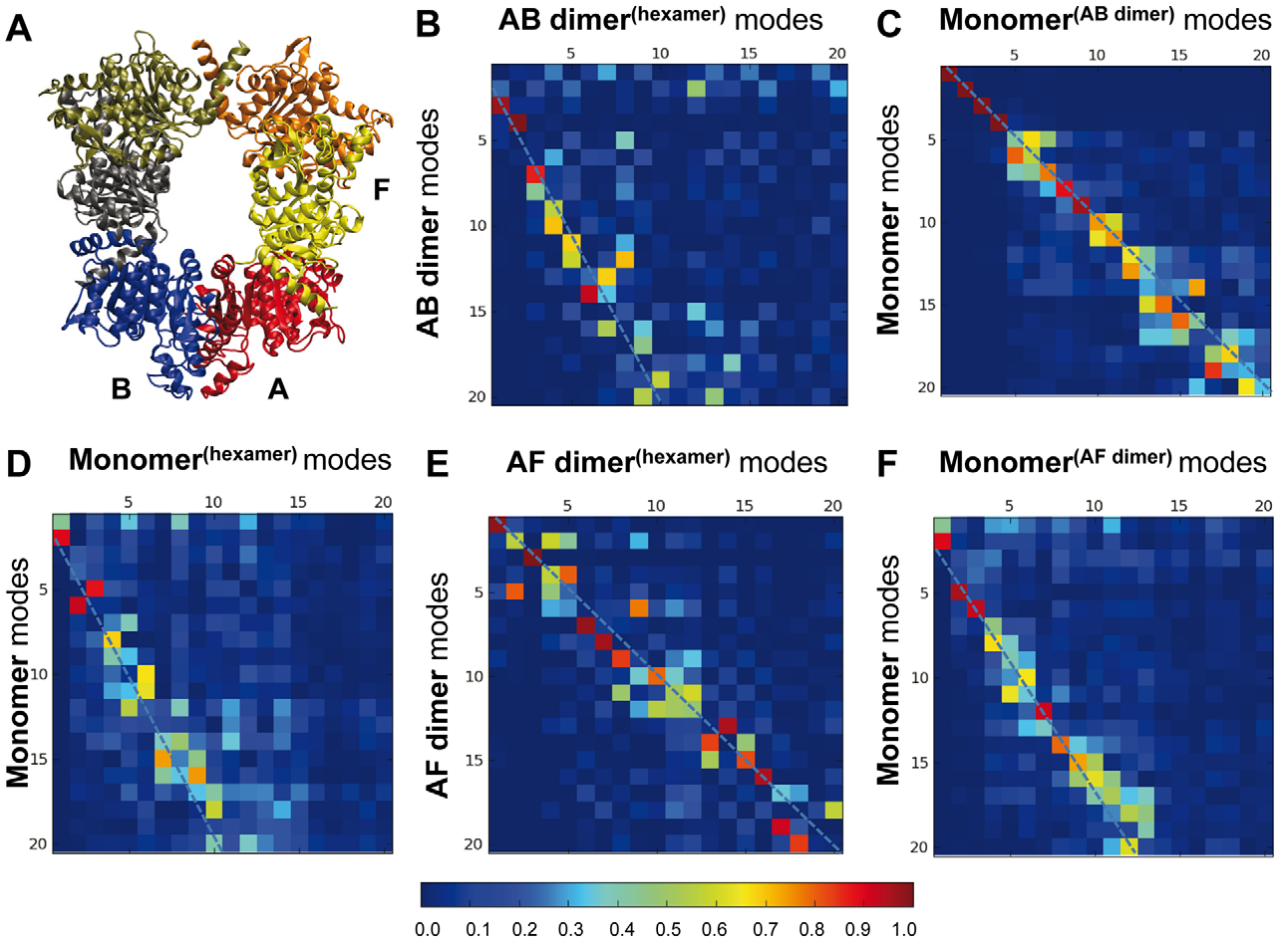
Comparison of the global dynamics of *Tm*NAGK monomers in the hexamer with those of the isolated monomeric and dimeric components. (A) Cartoon representation of *Tm*NAGK, illustrating the packing of the individual subunits, resulting in two different types of intersubunit interfaces represented by those in the AB and AF dimers. (B)-(F) Overlaps between the 20 slowest modes of different building blocks (monomer, AB and AF dimers) and the hexamer, as labelled in the individual heat maps. In all five maps, the weighted linear fit to overlaps above 0.5 is shown by the dashed line.

On the basis of the X-ray crystallographic structure, the hexameric arrangement of *Tm*NAGK is considered to be a trimer of *Ec*NAGK-like dimers [30], herein called the AB dimer (see Figures 1B and 4A). The dimeric scaffolds are interlaced by a mobile N-terminal helix, not present in the dimeric *Ec*NAGK, and organized with a ring shape. An alternative dimeric building block being considered is the one constituted by the two monomers that interlink two adjacent AB dimers, herein called the AF dimer (see Figure 4A). In the present study, we have compared the 20 lowest-frequency modes of the hexamer with those of the monomeric subunit and the two different dimeric building blocks.

The results are presented in the panels B–F of Figure 4. In each panel, the *x*-axis refers to the modes observed in the oligomer (hexamer or dimer), and the y-axis refers to those intrinsically accessible to the components (dimers or monomers) that make these oligomers, e.g., panel B compares the global modes of the AB dimer in the hexamer (x-axis) to those accessible to the AB dimer itself when examined in isolation (y-axis). The comparative examination of these maps discloses two distinctive patterns: panels C and E reveal the conservation of global modes, in general, between the entities that are being compared, while panels B, D and F reveal that about ½ of the modes accessible to the substructures when examined in isolation are *not* represented in the assemblies. This behavior is clearly seen, and quantified, by the dashed lines on the maps, which represent a linear fit by weighted least squares regression to the entries that exhibit a correlation of 0.5 of higher. The dashed line in the former groups lies along the diagonal (slope -1.04 and -1.01 in the respective panels C and E), whereas in the latter case, the slope varies as -1.81 (panel B), -1.72 (D) and -1.44 (F).

Let us first examine the 1^st^ group more closely: panel C essentially tells us that the monomers participating in the AB dimer maintain in the dimer their intrinsic dynamics favored by their monomeric architecture. As to panel E, it simply reflects that AF dimer in the hexamer behaves practically in the same way as in the isolated AF dimer, indicating that multimerization does not alter the global dynamics favored by the AF dimeric structure. In other words, the *Tm*NAGK hexamer exploits the intrinsic dynamics of the AF dimer; and likewise, the AB dimer takes advantage of the structure-encoded dynamics of its monomers. Notably, the top four modes are conserved in this case with a correlation of more than 0.95. This is in agreement with the high conservation of the monomer dynamics in the *Ec*NAGK dimer, as pointed out in Figure 3A, given the structural and dynamical similarities [34] between the AB dimer and *Ec*NAGK.

We now turn our attention to the 2^nd^ group. Here we see the dimer AB in the hexamer which is unable to sample several modes that are accessible to the same dimer in isolation (panel B). Thus, the environment provided by the hexamer constrains the intrinsic dynamics of the AB dimer. Why is the AB dimer rigidified in the hexamer? We note that in the hexamer, these *Ec*NAGK-like (AB) dimers make close, interlacing interactions with the adjacent dimer by swapping their *N*-terminal helices and also making contacts with the *C*-domain, i.e. the interactions of AB-type dimers with the adjacent dimer through the AF interface impose topological constraints that impair several modes in the hexamer (panel B). Likewise, the monomer in the hexameric environment is more restricted than the isolated monomer, such that many modes accessible to the isolated monomer cannot be effectuated in the hexamer (panel D). Given the different degree of conservation of the dynamics of the AB and AF dimers within the hexamer (panels B and E), we can add a complementary perspective to the structural view of *Tm*NAGK as a trimer of *Ec*NAGK-like dimers. The stronger conservation of the dynamics of the AF dimer supports a dynamical view of *Tm*NAGK as a trimer of AF-like dimers.

Finally, it is worth pointing out that the surface area of the AF interface (1186 Å^2^) is slightly smaller than that of the AB interface (1381 Å^2^) [30]. This might suggest that the monomeric modes would be more severely constrained in the AB dimer, but this does not hold true as explained above. The small difference in the surface area is therefore not sufficient to explain the observed behavior. The major determinant of accessible global motions is not the surface area but the topology of the interfacial contacts, or the overall shape/architecture of the dimer. In the present case, the overall architecture of the hexamer selectively hinders a number of global modes accessible to the AB dimer, while those of the AF dimer are mostly preserved. It is widely accepted that the size of the interface is closely linked to the thermodynamic stability of the oligomer [25], [55]. The dynamics of the oligomer, on the other hand, is suggested by the present analysis to be predominantly controlled by the quaternary arrangement and contact topology of the subunits.

### New modes of motion and cooperativity

The results discussed above focus on the preservation or the obstruction of the global motions of the subunits upon oligomerization. Nevertheless, in many cases, oligomeric proteins are subject to cooperative processes that regulate the biological activity. This raises the question whether such cooperative processes are linked to new modes of motion unique to oligomeric arrangement.

*Tm*NAGK is cooperatively inhibited by arginine in contrast to the dimeric *Ec*NAGK and *Pf*CK, which do not exhibit an allosteric regulation. The available X-ray crystallographic structure of *Tm*NAGK represents the T state of the enzyme, which is bound to arginine. The apo form of the enzyme (R state) has not been structurally resolved, but the X-ray structure of the same enzyme from *Pseudomonas aeruginosa* (*Pa*NAGK) serves as a suitable model for the R state on the basis of sequence and structural similarities [30]. Taking into account that the transition of *Tm*NAGK between the R and T states is intimately linked to its allosteric regulation, those modes of motion that favor this conformational change will be the most functional. Therefore, the cumulative overlap of the lowest modes with the deformation vector between the R and T states has been calculated. Given that the T and R states correspond to proteins with different sequences, we have structurally aligned the two structures with DALI [56] and used the subsystem/environment coupling method (see *Methods*) to compute the ANM modes of *Tm*NAGK, considering as subsystem those residues of *Tm*NAGK structurally aligned to *Pa*NAGK. Likewise, the deformation vector was calculated for the structurally aligned residues.

**Figure 5:**
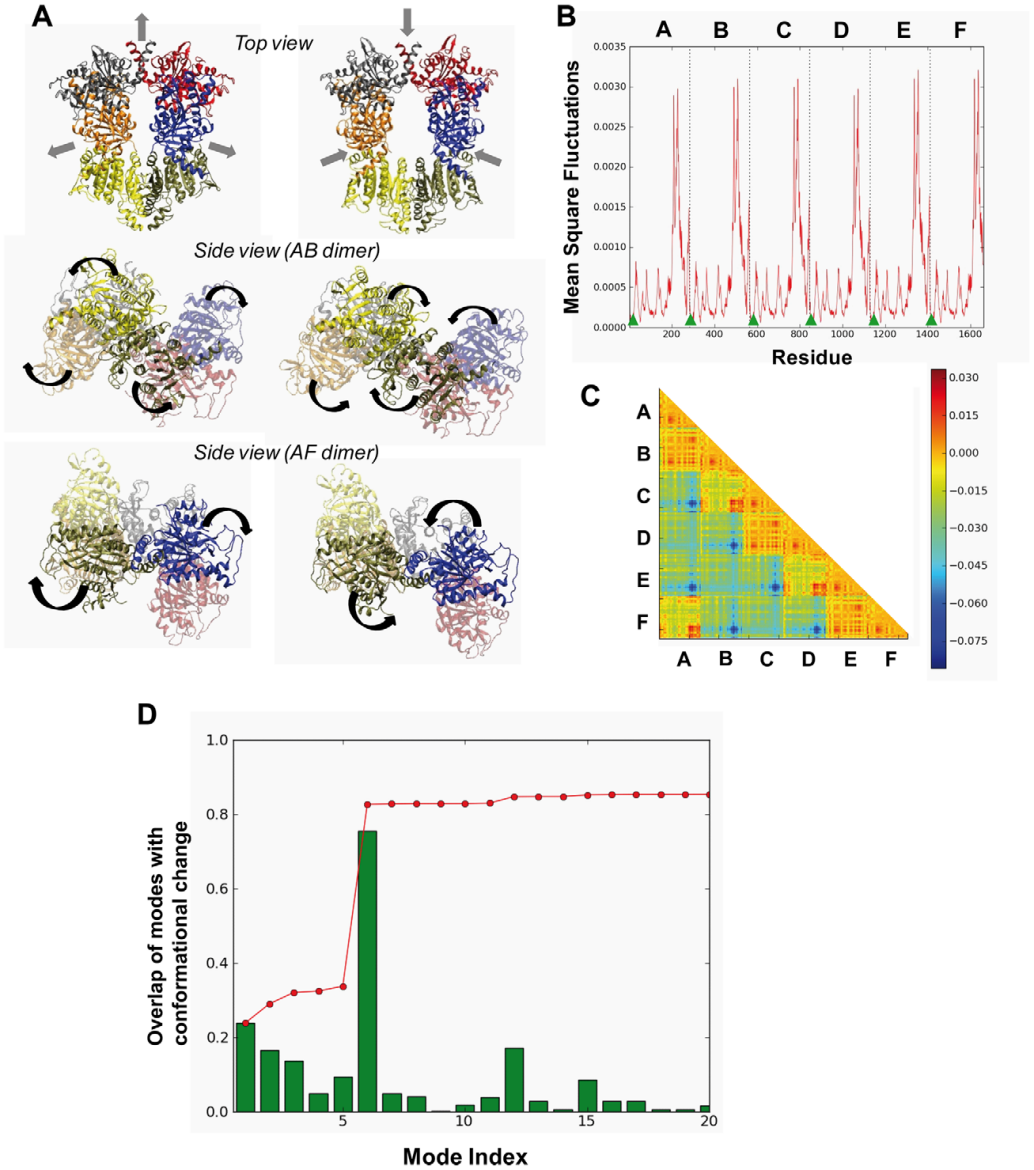
The cooperative mode of motion that enables the T→R transition of hexameric *Tm*NAGK. (A) Schematic description of the *Tm*NAGK mode 6 which yields a remarkably high overlap with the structural change involved in the T→R transition of the enzyme. Two structures have been generated using Eq.3. The top view of the deformed structures shows the opening/closure of the ring. The arrows show the direction of motion. The side views of the AB and AF dimers show a rotational movement of both dimers that make the hexameric ring flatter when it opens. See Video S5 for better visualization. (B) Mean-square displacements of residues in the 6^th^ mode. Hinge sites are indicated by solid triangles. (C) Inter-residue distance variation map in mode 6. Blue/red/orange entries refer to distances that decrease/increase/remain unchanged. If the inter-residue distances within a given subunit remain constant, this indicates a rigid-body motion of the subunit (see Eq. 5). (D) Overlap of individual *Tm*NAGK modes with the allosteric change in structural coordinates between the T and R states. *Red line*: cumulative overlaps CO(*m*) between ANM modes and the experimentally observed conformational transition between R and T states (see Eq. 4), calculated for the 20 lowest-frequency modes. *Green bars*: overlap of each mode. This subset of 20 modes accounts for 85% of the conformational change, predominantly contributed by the 6th mode (overlap of 75%).

Strikingly, a single non-degenerate mode (6^th^) accessible to *Tm*NAGK is found to describe 75% of the R↔T deformation (see Figure 5D showing the cumulative overlap). A deeper analysis of this mode can shed light on the structural origin of the functionality of this enzyme. The aim is to ascertain whether this mode arises from the intrinsic dynamics of the subunits or is acquired in the hexameric state. Mode 6 is an expansion/contraction of the ring, accompanied by cooperative rotational and twisting motions of each monomer (see Video S5). The axis of rotation goes through each AF interface (Figure 5A) and performs an almost rigid rotation of the *Ec*NAGK-like dimers (Figure 5C). Residues close to these axes of rotation form minima in the mode fluctuations profile (Figure 5B) and belong to the AF interface. The axis involves a part of the *N*-terminal helix (6–20) of chains A and F, where the two helices interact tightly. Indeed, this interface stabilizes the hexameric arrangement and no NAGK dimer has been structurally characterized with an AF-like interface. The AF interface is unique to the hexameric arrangement.

As shown in Figure 4, the hexamer dynamics is affected by the intrinsic dynamics of the component subunits. Therefore, mode 6 could be associated with particular global modes accessible to the AB and/or AF dimers. We have examined the inter-residue distance variations maps induced by the low-frequency modes of the isolated AB and AF dimers to explore this possibility. AF dimer proves to be the major source of the rigid body movements of monomers observed in the hexamer (see Videos S6 and S7). The distance variation maps of the 1^st^ and 4^th^ modes of the AF dimer (Figure S1) illustrate that the internal motions within a given subunit are negligible, but the relative movements between the two subunits are significant. The AF interface, thus, emerges as a key mechanical region that confers to the two linked subunits suitable flexibility to undergo functional changes in their relative orientations. This dynamic feature of the AF interface, whose size is smaller than the AB interface, is in accord with Hubbard and co-workers [57], who stated that those interfaces that are not optimally packed may confer functional mobility to the oligomer. This inherent dynamical ability of the AF interface is therefore exploited in the hexameric arrangement to couple the rigid-body movements of the subunits, complementing their intrinsic internal dynamics.

### Communication across the structure

**Figure 6:**
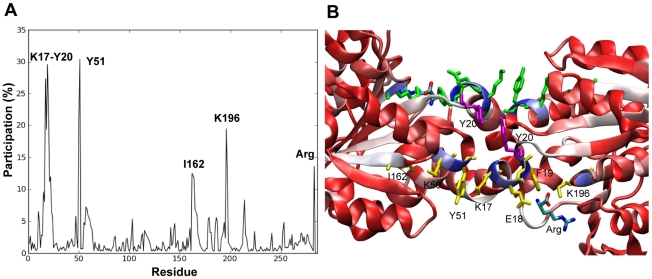
Communication pathways at the AF interface. (A) Percentage of communication pathways in which a given residue is on-pathway. (B) Color coded-ribbon diagram of the AF interface. The color code refers to the participation of the residues in the located communication pathways (the participation increases from red to blue).The main communication pathways across the interface are colored in green, yellow and violet, and the residues on-pathway are labeled.

The topology of the AF interface appears to be evolutionary selected to provide two essential features for the functionality of the enzyme: (1) flexibility to allow for the cooperative reorientations of the dimers, which is inextricably linked to allostery, and (2) thermodynamic stability of the whole hexamer. Taking into account the crucial role of the AF interface and with the aim of providing further insights into the allosteric regulation of this enzyme, we considered the maximum likelihood pathway (MLP) for each combination of pairs of residues (endpoints) belonging to the respective chains A and F, and evaluated the fractional occurrence of each residue in the ensemble of MLPs (see Methods). Figure 6A displays the percent occurrence of each residue, which also provides a measure of the relative allosteric potential of the residues. Peaks are observed at K17, E18, F19, Y20, K50 and Y51 (ribbon diagram color-coded from blue (peaks) to red (minima) in Figure 6B). The significance of this first set in allosteric communication could be anticipated due to their location at the tightest part of the AF interface and proximity to the arginine inhibitor (Figure 6B). However, our approach helps to identify other distal residues important for the communication, which behave as hubs. In particular, K196 and I162 channel most of the pathways to the AF interface via interactions with F19 (and the arginine inhibitor) and K50, respectively.

The communication across the AF interface can be summarized namely by two symmetric pathways distinguished by the MLP analysis: I162_A_ → K50_A_→ Y51_A_→ K17_F_ → E18_F_ → F19_F_ → K196_F_ and its counterpart I162_F_ →…→ K196_A_ (colored yellow and green in Figure 6B). Aromatic residues tend to be favored at protein interfaces [25], and in this case, F19 and Y20 play a critical role. Not surprisingly, F19 is highly conserved among arginine-sensitive NAGKs [30] and, together with Y20 (violet in Figure 6B), it establishes an efficient communication pathway of the form F19_(A/F)_→ Y20_(A/F)_→ Y20_(F/A)_→ F19_(F/A)_.

### Differences in the dimer organization point to different functional mechanisms: *Ec*NAGK vs *Ec*UMPK

**Figure 7:**
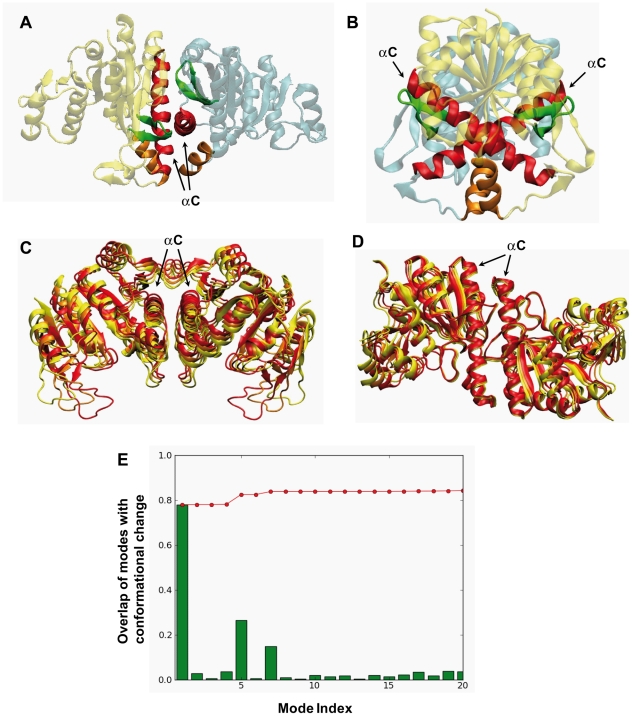
Comparison between *Ec*NAGK and *Ec*UMPK dimmers. (A) and (B) Ribbon representations of two perpendicular views of the *Ec*NAGK dimer. A different color is used for each subunit. Secondary structure elements building the intersubunit surface are colored differently (helices αC in red, β9-β10 hairpins in green and helices αB in orange). (C) and (D) Ribbon representations of two perpendicular views of the *Ec*UMPK dimer. Different conformations along the 1^st^ ANM mode are generated with Eq. 3 (s = -20 in red , s = 0 in orange and s = 20 in yellow). See Video S8 for better visualization. (E) Comparison of *Ec*UMPK dimer modes with the allosteric conformational change observed in the GTP-bound form. *Red line:* cumulative overlaps CO(*m*) between ANM modes and the experimentally observed conformational transition between the UDP- and GTP-bound states (Eq. 4 in *Methods*), calculated for the 20 lowest-frequency modes. *Green bars:* overlap of each mode with the conformational change. This subset of 20 modes accounts for 84% of the conformational change, being predominantly contributed by the 1^st^ mode.

The structure of the monomeric subunit of *Ec*NAGK is preserved among all family members, but the assembly geometry is less conserved. The arrangement of the monomeric subunits of NAGKs and CKs is strikingly similar, as shown above, but has significant differences with the assembly of UMP Kinases. Structurally, UMPKs are trimers of dimers in which the two helices that build the intersubunit surface of each dimer are parallel (Figure 7C and D), whereas in NAGK (and CK) these helices at the interface make an angle of ∼65° (Figure 7A and B). To our knowledge, a clear functional reason for this difference in monomer-monomer packing has not been reported so far. Although this difference has been argued to be necessary for hexameric assembly [58], there might be another functional reason since *Tm*NAGK is an example of a hexameric assembly that selectively adapts the *Ec*NAGK-like dimer packing (AB dimer). Here we compute the ANM modes of the UPMK dimer from *Escherichia Coli* (*Ec*UMPK) in order to examine whether such a difference in packing geometry gives rise to significant changes in the global dynamics.

The first mode of motion of the isolated *Ec*UMPK dimer entails a rotational rigid-body movement with respect to an axis across the αC helices (Figure 7, panels C and D, and Video S8). The anticorrelated motion of both subunits leads to an opening/closure movement of the whole dimer. This is in sharp contrast to the *Ec*NAGK dimer dynamics, whose low-frequency modes do not exhibit rigid-body movements of the subunits. Does this dynamic feature of the *Ec*UMPK dimer play a functional role?

**Figure 8:**
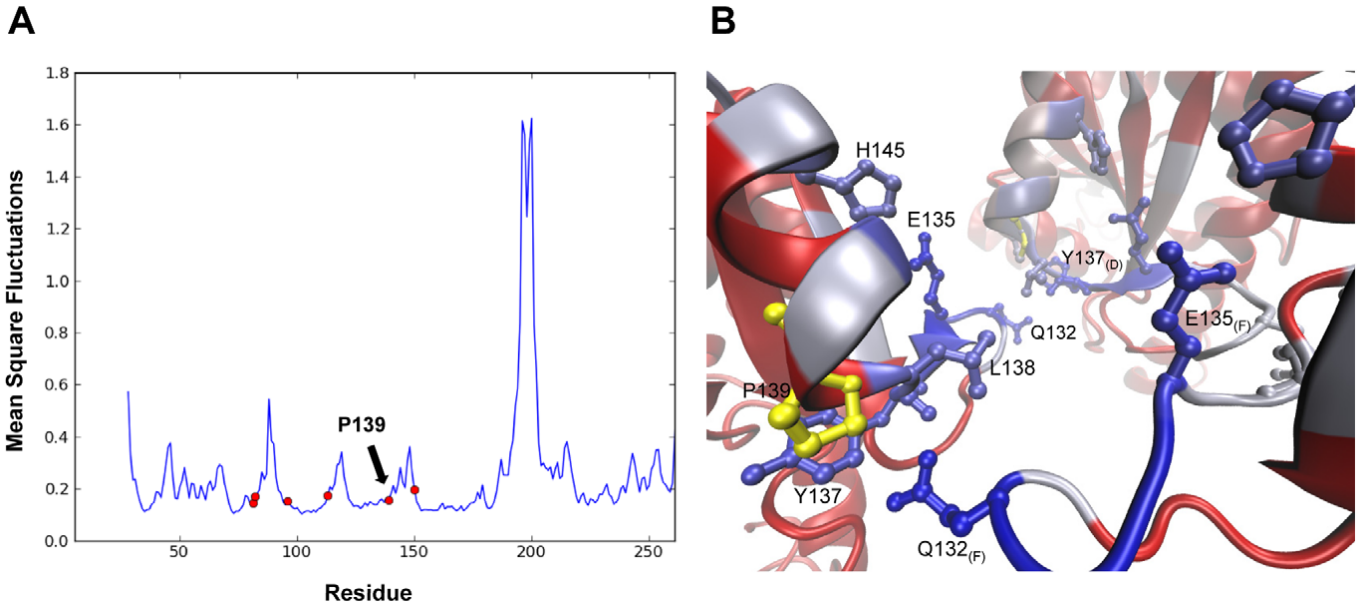
Collective dynamics and signal propagation in *Mt*UMPK. (A) Mobility profile obtained by ANM computed for all 3*N*-6 normal modes of the hexamer. The fluctuations of only one monomer are shown. Red dots correspond to those positions that were mutated in the study of Labesse and co-workers [60] (B) Color coded ribbon diagram of the interface. The color code (same as in Figure 6B) refers to the participation of the residues in the located communication pathways. P139 (shown in yellow) does not directly participate in intersubunit communication but highly constraints the neighboring residues Y137 and L138 that play a key role in allosteric signaling.

Gilles and co-workers determined the X-ray crystal structure of *Ec*UMPK complexed with GTP (PDB code 2VRY) [59], which is an allosteric activator, and characterized a functional conformational change. They argued that GTP induces a rearrangement of the quaternary structure that involves a rigid-body rotation of 11° that opens the UMPK dimer. Strikingly, the first ANM mode predicted for the UDP-bound dimer describes the structural transition between the UDP- and GTP-bound forms. The overlap is outstandingly high (0.78) (see Figure 8E for cumulative overlap). Moreover, it is worth pointing out that we have checked that this mode of motion is totally conserved in the hexamer (see Figure S2).

Why does the different assembly in the UMPK dimer give rise to a normal mode with a rigid-body character not present in *Ec*NAGK? In UMPK the interface between the monomers is constituted mainly by two long parallel helices (αC) able to build a rotational axis that promotes an *en bloc* motion of both subunits. In contrast, the crossed orientation of the helices of NAGK (∼65°) and the presence of other intersubunit contacts (B-helices and β9–β10 hairpins) hinders a rigid-body rotation of the two subunits. This suggests that the unique dimeric assembly of UMPK gives rise to a particular soft mode not present in other AAK family members. This example further indicates that the design of the interfacial contact topology and oligomerization geometry is crucial in defining the functional mechanisms of oligomers.

### Importance of spatial constraints in the allosteric regulation of UMPK

In some cases, a single residue may significantly affect the contact topology at the interface and, thus, the allosteric regulation. This has been explored in the context of the UMPK analogue from *Mycobacterium tuberculosis* (*Mt*UMPK), for which crystallographic and site-directed mutagenesis studies have been recently conducted [60]. The X-ray structure of *Mt*UMPK bound to GTP shows striking similarities to *Ec*UMPK structure. Notably, this similarity is extended to their global motions: the lowest frequency ANM modes of the two structures exhibit an overlap of 0.97. Given that the global modes of motion are fully determined by the overall shape of the protein, local perturbations are indeed unlikely to affect the low-frequency modes.

Site-directed mutagenesis studies, on the other hand, show the importance of some residues in both the activity and the cooperativity of the enzyme. Among them, P139 was pointed out to to be a key residue in the allosteric regulation of the enzyme. P139 is located close to the trimeric interface where three GTP molecules are bound. What is the dynamical role of this residue? The mean-square fluctuations profile obtained with the ANM shows that P139 occupies a position close to a local minimum (a rigid part of the protein) (Figure 8A). Such regions usually play a key mechanical role for mediating collective changes in structure, and mutations at such positions may potentially affect the allosteric dynamics of the protein.

We have analyzed the importance of P139 in mediating the allosteric communication among subunits A, D and F, which build one of the two trimeric interfaces where three GTP molecules are bound. We computed the communication pathways between GTP binding residues (starting from subunit A and ending at subunits D and F) and the percent contribution of each residue to MLPs, as done for *Tm*NAGK. Figure 8 shows the trimeric interface color-coded according to the percent contribution in the same way as in Figure 6B. We note that the participation of P139 (in yellow) to these pathways is minimal (note the red color in the backbone), but the adjacent residues Y137 and L138 are important mediators of inter-subunit communication via interactions with Q132.

This analysis suggests that the importance of P139 lies in constraining the orientation of nearby residues Y137 and L138 involved in inter-subunit signal propagation. The fact that this residue is highly restricted position in the global mode profile emphasizes its role in constraining the neighboring residues in a precise orientation pre-disposed to enable inter-subunit communication. The experimentally tested mutants (P139A, P139W and P139H) all showed a diminished allosteric regulation, but to different extents [60]. Further simulations at atomic scale might help explain the relative sizes of the effects induced by these mutations, but this is beyond the scope of the present work. It might be interesting to experimentally test the effect of mutations at L18, Y137 and Q132, since these residues emerge here as key elements enabling inter-subunit communication and they are distinctly restricted in the collective dynamics (Figure 8A) despite the relatively low packing density at the interface.

To summarize, the present study reveals several dynamic features of oligomeric proteins by means of an ENM analysis of family members with different degrees of oligomerization. A common dynamic feature of the oligomers presented here is the conservation of the inherent dynamics of their monomeric or dimeric building blocks. The way these blocks are assembled in different oligomers confers different types of collective mechanisms unique to particular oligomerization geometries. Here are the main observations:

1. The dimeric *Ec*NAGK and *Pf*CK conserve to a high extent those normal modes of the monomers which involve minimal conformational rearrangements at the intersubunit interface.
2. The topology of the interface in *Pf*CK provides the protruding subdomains of the component subunits with remarkably high mobility, which apparently enhances the affinity for binding the carbamate substrate and for excluding other carbamate analogues that are more abundant, as suggested by recent experiments [37].
3. The *Tm*NAGK hexamer has two different types of interfaces (AB and AF) that provide different dynamic properties to the hexamer. The AF interface provides the hexamer with the ability to perform *en bloc* motions that cooperatively engage all six subunits, in contrast to the AB interface that enjoys an internal flexibility relevant to the opening of the substrate binding site. The concerted movements of the six subunits coupled to the internal motion of the subunits give rise to a normal mode (mode 6, Figure 5) intimately linked to the allosteric transition of the hexameric enzyme.
4. The ability of *Tm*NAGK to enable allosteric signaling has been studied by means of a Markov model of network communication. The MLPs connecting residues of chains A and F suggest that some residues of the interlaced N-terminal helices, which build the AF interface (e.g., K17, E18, F19 and Y20) are distinguished by their high allosteric potential. Notably, these residues coincide with the key mechanical sites (global hinges) that mediate the cooperative mode of motion.
5. The different assembly of the subunits in the *Ec*UMPK dimer, with respect to *Ec*NAGK, gives rise to rigid-body movements of the subunits that are necessary for the allosteric regulation of *Ec*UMPK. The mutual disposition of the two long helices that build the interface in either enzyme proves to be crucial for favoring functional dynamics. Interestingly, the experimentally observed allosteric switch mechanism of UMPK is closely reproduced by a single mode (ANM mode 1; Figure 7E), in support of the functional significance of the collective motions uniquely defined by the dimeric architecture.
6. In parallel with the observations made for *Tm*NAGK allosteric communication, a series of residues highly restricted in the collective dynamics of *Mt*UMPK play a key role in enabling intersubunit communication. P139 plays a structural role by introducing backbone constraints that precisely constrain nearby residues' side chains in orientations pre-disposed to optimal binding of GTP and inter-subunit communication. The significance of P139 in enabling allosteric communication is consistent with site-directed mutagenesis data [60].

In summary, the oligomers in the examined AAK family appear to selectively exploit the inherent dynamic abilities of its components, on the one hand, and favor coupled movements of intact subunits, on the other, to effectively sample cooperative movements (soft modes) that enable motions required for substrate binding and efficient allosteric responses. The architecture of the interfaces and the assembly geometry play an essential role in defining the most easily accessible (or softest) modes of motion, which in turn, are shown to be relevant to the functional mechanisms of the different oligomers, being presumably optimized by evolutionary pressure.

## Methods

### Anisotropic Network Model (ANM)

The low-frequency modes described by the NMA of different ENM variants [40], [61]–[64] have proven to be robustly determined by the overall fold [7], [65], [66] and provide a consistent description of the conformational space most easily accessible to the protein [67]. Among them, we use here the most broadly used model, the anisotropic network model (ANM) [40], [41]. In the ANM, the network nodes are located at the C^α^-atoms' positions, and pairs of nodes within close proximity (a cutoff distance of 15 Å, including bonded or non-bonded pairs of amino acids [41]) are connected by springs of uniform force constant γ. The interaction potential of the molecule is given by
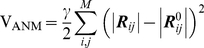
(1)


where *M* is the number of springs, and |***R*** *_ij_*|-|***R*** *_ij_^0^*| is the inter-residue distance with respect to the equilibrium (crystal) structure. The second derivatives of V_ANM_ with respect to residue displacements yield the 3Nx3N Hessian matrix **H**, the eigenvalue decomposition of which yields 3N-6 nonzero eigenvalues λ*_k_* and eigenvectors ***u*** *_k_* corresponding to the frequencies (squared) and shapes of the normal modes of motion accessible to the examined structure. Numbering of modes in this work starts from the first mode with a nonzero eigenvalue.

The cross-correlation between the displacements of residues *i* and *j*, contributed by mode *k* scales as
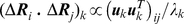
(2)


where the subscript *ij* designates the element of the matrix in square brackets. For *i*  = *j*, equation (2) reduces to the square displacement of residue *i* in mode *k*. Clearly, lower-frequency modes (smaller *λ_k_*) drive larger-amplitude motions.

### Generation of large-amplitude conformational changes

Conformations sampled upon moving along mode *k* are generated using
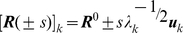
(3)


where ***R^0^*** is the 3*N*-dimensional vector representing the initial coordinates of all residues and *s* is a parameter that rescales the amplitude of the deformation induced by mode *k*. The movies S1-S8 in the Supporting Information are generated using this equation with a series of different *s* values for selected modes of examined proteins.

### Comparison of experimental conformational changes with normal modes

The degree of overlap between a conformational change *Δ* ***r*** observed by X-ray crystallography and the structural change predicted by the ANM to take place along mode *k* is quantified by (***Δr*** **·** ***u*** *_k_*)/|***Δr***|. Here *Δ* ***r*** is the *3N*-dimensional difference vector between the α-carbon coordinates of two different forms resolved for the same protein under different conditions (e.g., substrate-bound and -unbound forms of enzymes, or inward-facing or outward-facing forms of transporters). The cumulative overlap CO(*m*) between *Δ* ***r*** and the directions spanned by a subset of *m* modes is calculated as 
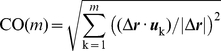
(4)


CO(*m*) sums up to unity for *m* = 3*N*-6, as the eigenvectors form a complete orthonormal set of basis vectors in the 3*N*-6 dimensional space of internal conformational changes (see Figures 5D and 7E)

### Subspace overlap

The similarity between the conformational spaces described by two subsets of *m and n* modes, ***u*** *_k_* and ***v*** *_l_*, evaluated for two different systems can be quantified in terms of a double summation over squared overlaps as in Eq. 4, among all *m*x*n* pairs of modes (divided by *m* or *n*, depending on the reference set). The overlap *O*(***u*** *_k_*,***v*** *_l_*,) between the pairs of modes ***u*** *_k_* and ***v*** *_l_* calculated for different systems (e.g., Figure 3) is given by the inner product of the eigenvectors, i.e., 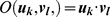
(5)


Note that *O*(***u*** *_k_*,***v*** *_l_*,) is equal to the correlation cosine between the two N-dimensional vectors, since the eigenvectors are normalized.

### Distance variation maps

The change in a given inter-residue distance |***R*** *^0^_ij_*| induced by a given mode *k*, 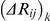, is given by the projection of the deformation induced by the *k^th^* mode onto the normalized distance vector, scaled by the inverse frequency, 
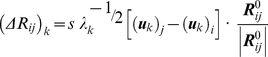
(6)


Here (***u*** *_k_*)*_i_* designates the *i^th^* super element (a 3D vector) of ***u*** *_k_*, and describes the relative displacement of the *i^th^* residue (*x*-, *y*-, and *z*-components) along the *k^th^* mode direction.

### Communication pathways

Inter-residue communication has been suggested to play a key role in allosteric regulation and enzymatic catalysis [68], [69], and has been the subject of many computational studies [48], [70]–[72]. Here we use a Markov model of network communication [73], [74] to identify communication pathways. The interactions between residue pairs connected in the ANM are defined by the affinity matrix **A**, whose elements are *a_ij_ = N_ij_/(N_i_ N_j_)^½^* where *N_ij_* is the number of atom-atom contacts between residues *i* and *j* based on a cutoff distance of 4 Å, and *N_i_* is the number of heavy atoms belonging to residue *i*. The density of contacts at each node *i* is given by 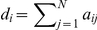.The Markov transition matrix ***M*** = {*m_ij_*}, where *m_ij_* = *a_ij_*/*d_j_*_,_ determines the conditional probability of transmitting a signal from residue *j* to residue *i* in one time step [73]. We define –log(*m_ij_)* as the corresponding ‘distance’. The maximum-likelihood paths (MLPs) for signal transfer between two end points are evaluated using the Dijkstra's algorithm [73]. In order to identify the residues that play a key role in establishing the communication between pairs of subunits, we considered the communication between all pairs of residues belonging to the two subunits of interest. In the application to the communication between the A and F subunits of *Tm*NAGK (Figure 6), an ensemble of *N* ^2^ = 282^2^ combinations of residue pairs (endpoints) have thus been considered (each chain consists of *N* = 282 residues). For each pair, we evaluated the MLP and thus determined the series of residues taking part in the MLP. To quantify the contribution of a given residue to intersubunit communication, we counted the occurrence of each residue in the complete ensemble of MLPs. Figure 6, panel A displays the resulting curve, peaks indicating the residues that make the largest contribution.

### NMA of a subsystem coupled to a dynamic environment

In many applications the dynamics of a part of the protein (subsystem, S) may be of interest in the context of its environment (E). The Hessian of the whole system is conveniently partitioned into four submatrices [75], [76]:
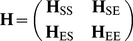
(7)


where **H**_SS_ is the Hessian submatrix for the subsystem, **H**_EE_ is that of the environment and **H**_SE_ (or **H**_ES_) refers to the coupling between the subsystem and the environment. Inasmuch as the environment responds to the subsystem structural changes by minimizing the total energy, the effective Hessian for the subsystem 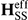 coupled to the environment is 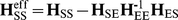
(8)


This approach has been advantageously employed in determining potential allosteric sites [77] and locating transition states of chemical reactions [78]. It will be used below in conjunction with the ANM for assessing the effect of oligomerization on the dynamics of monomeric and/or dimeric components (subsystem).

### Structural data

We examined four enzymes belonging to the AAK family (Figure 1): *Ec*NAGK (dimer), *Tm*NAGK (hexamer), *Pf*CK (dimer) and *Ec*UMPK (hexamer). To this aim, we use the X-ray structures of *Ec*NAGK in the open state (PDB code: 2WXB), the arginine-bound *Tm*NAGK (PDB code: 2BTY), the ADP-bound *Pf*CK (PDB code: 1E19) and the UDP-bound *Ec*UMPK (PDB code: 2BND).

All diagrams of molecular structures have been generated using VMD [79].

## Supporting Information

Figure S1 **Distance variation maps of the 1^st^ and 4^th^ modes of the AF dimer.** Blue positions indicate that the distance between two residues decreases, and a red position that it increases. If the inter-residue distances within a given subunit remain constant, this indicates a rigid-body motion of the subunit. See Videos S6 and S7 for better visualization of these two normal modes.
(TIF)

Figure S2 **Comparison of the global dynamics of the dimeric component of** ***Ec*** **UMPK in the hexamer with that of the isolated dimeric component.** Overlaps between the 20 slowest modes of the dimer and hexamer are labelled in the heat map. The AB dimer is highlighted in the ribbon diagram of *Ec*UMPK and the rest of the hexamer (the environment) is shadowed. The structure is colored by chains. The first mode of the dimer is expressed by two modes within the hexamer (the overlap with hexameric modes 1 and 3 is 0.73 and 0.58, respectively). The dynamic properties of the dimer are remarkably well conserved in the hexamer as given by a subspace overlap of 0.95 of the 20 lowest-frequency modes.
(TIF)

Table S1 **Overlap between the eight lowest frequency modes of the isolated** ***Ec*** **NAGK monomer and the** ***Ec*** **NAGK monomer within the dimmer.**
(DOC)

Table S2 **Overlap between the eight lowest frequency modes of the isolated** ***Pf*** **CK monomer and the** ***Pf*** **CK monomer within the dimmer.**
(DOC)

Video S1 **Symmetric substrate binding mode of motion of dimeric** ***Ec*** **NAGK (ANM mode 5).**
(WMV)

Video S2 **Asymmetric substrate binding mode of motion of dimeric** ***Ec*** **NAGK (ANM mode 4).**
(WMV)

Video S3 **Symmetric substrate binding mode of motion of dimeric** ***Pf*** **CK (ANM mode 3).**
(WMV)

Video S4 **Asymmetric substrate binding mode of motion of dimeric** ***Pf*** **CK (ANM mode 4).**
(WMV)

Video S5 **Allosteric mode of motion of hexameric** ***Tm*** **NAGK (ANM mode 6).**
(WMV)

Video S6 **Mode of motion of the isolated AF-type dimer of** ***Tm*** **NAGK (ANM mode 1).**
(WMV)

Video S7 **Mode of motion of the isolated AF-type dimer of** ***Tm*** **NAGK (ANM mode 4).**
(WMV)

Video S8 **Allosteric mode of motion of the dimeric component of** ***Ec*** **UMPK (ANM mode 1).**
(WMV)
