# Supplementary figures and images for: Changes in Dynamics upon Oligomerization Regulate Substrate Binding and Allostery in Amino Acid Kinase Family Members
Source: PLoS Comput Biol. 2011 Sep 29;7(9):e1002201. doi: 10.1371/journal.pcbi.1002201 (PMC3182869; doi:10.1371/journal.pcbi.1002201)

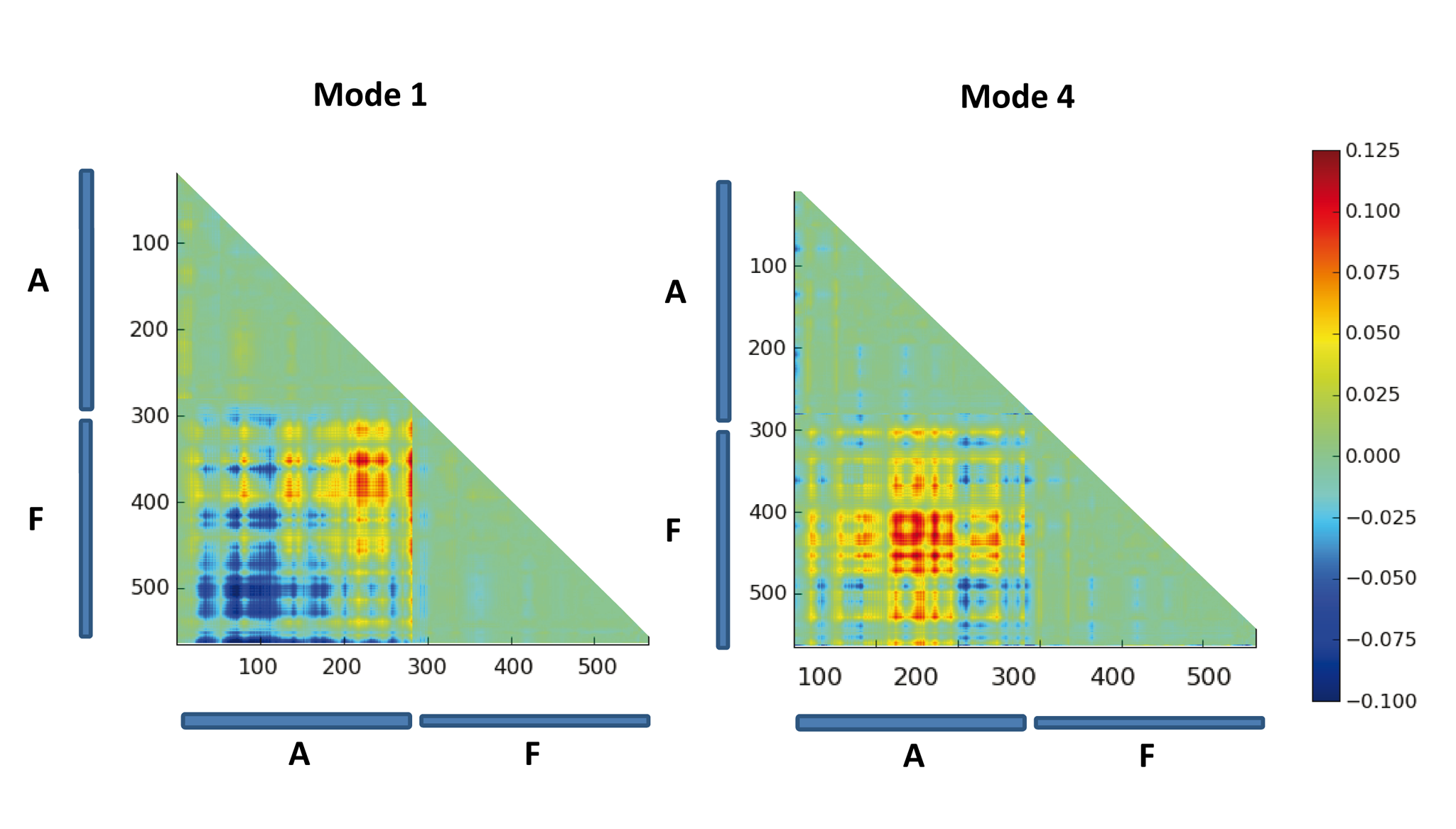

Supplement: Figure S1 — Distance variation maps of the 1st and 4th modes of the AF dimer. Blue positions indicate that the distance between two residues decreases, and a red position that it increases. If the inter-residue distances within a given subunit remain constant, this indicates a rigid-body motion of the subunit. See Videos S6 and S7 for better visualization of these two normal modes. (TIF) [file pcbi.1002201.s001.tif]

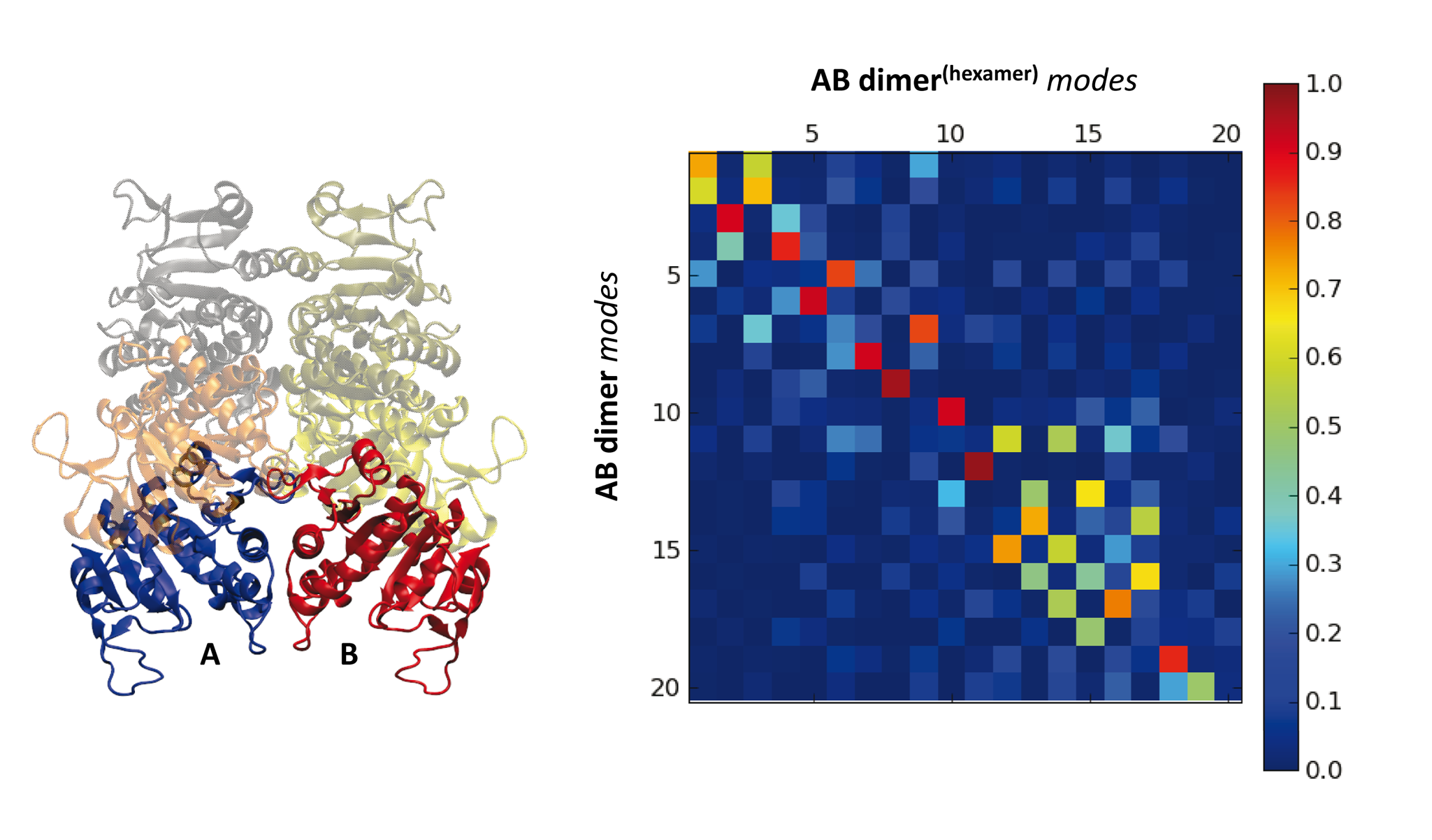

Supplement: Figure S2 — Comparison of the global dynamics of the dimeric component of Ec UMPK in the hexamer with that of the isolated dimeric component. Overlaps between the 20 slowest modes of the dimer and hexamer are labelled in the heat map. The AB dimer is highlighted in the ribbon diagram of EcUMPK and the rest of the hexamer (the environment) is shadowed. The structure is colored by chains. The first mode of the dimer is expressed by two modes within the hexamer (the overlap with hexameric modes 1 and 3 is 0.73 and 0.58, respectively). The dynamic properties of the dimer are remarkably well conserved in the hexamer as given by a subspace overlap of 0.95 of the 20 lowest-frequency modes. (TIF) [file pcbi.1002201.s002.tif]
