# Supplementary material for: Changes in Dynamics upon Oligomerization Regulate Substrate Binding and Allostery in Amino Acid Kinase Family Members
Source: PLoS Comput Biol. 2011 Sep 29;7(9):e1002201. doi: 10.1371/journal.pcbi.1002201 (PMC3182869; doi:10.1371/journal.pcbi.1002201)
Supplement: Supplementary file 2 [file pcbi.1002201.s003.doc]

		Ec NAGK monomer$^{(dimer)}$ modes							
		**1**	**2**	**3**	**4**	**5**	**6**	**7**	**8**
***Ec* NAGK monomer modes**	**1**	0.81	0.41	0.03	0.04	0.25	0.06	0.03	0.05
	**2**	0.48	0.81	0.19	0.06	0.12	0.11	0.10	0.03
	**3**	0.15	0.20	0.95	0.08	0.02	0.10	0.04	0.06
	**4**	0.11	0.17	0.11	0.92	0.28	0.01	0.02	0.03
	**5**	0.23	0.25	0.01	0.37	0.77	0.16	0.04	0.02
	**6**	0.07	0.17	0.19	0.10	0.07	0.72	0.47	0.07
	**7**	0.07	0.05	0.06	0.00	0.07	0.38	0.78	0.36
	**8**	0.03	0.02	0.07	0.04	0.20	0.45	0.24	0.62
