# Supplementary material for: Changes in Dynamics upon Oligomerization Regulate Substrate Binding and Allostery in Amino Acid Kinase Family Members
Source: PLoS Comput Biol. 2011 Sep 29;7(9):e1002201. doi: 10.1371/journal.pcbi.1002201 (PMC3182869; doi:10.1371/journal.pcbi.1002201)
Supplement: Supplementary file 3 [file pcbi.1002201.s004.doc]

		PfCK monomer(dimer) modes							
		**1**	**2**	**3**	**4**	**5**	**6**	**7**	**8**
***Pf*CK monomer modes**	**1**	0.73	0.34	0.23	0.12	0.12	0.11	0.02	0.02
	**2**	0.49	0.57	0.43	0.17	0.13	0.18	0.08	0.16
	**3**	0.45	0.08	0.03	0.00	0.25	0.00	0.01	0.11
	**4**	0.02	0.73	0.55	0.17	0.13	0.17	0.02	0.11
	**5**	0.03	0.01	0.25	0.17	0.28	0.03	0.71	0.34
	**6**	0.01	0.04	0.15	0.11	0.55	0.03	0.37	0.23
	**7**	0.05	0.03	0.22	0.47	0.38	0.04	0.18	0.04
	**8**	0.05	0.05	0.20	0.68	0.22	0.07	0.28	0.02
